# Molecular Mechanisms of Peritoneal Membrane Pathophysiology

**DOI:** 10.3390/biom12060757

**Published:** 2022-05-29

**Authors:** Sotirios G. Zarogiannis, Claus Peter Schmitt

**Affiliations:** 1 Department of Physiology, Faculty of Medicine, School of Health Sciences, University of Thessaly, BIOPOLIS, 41500 Larissa, Greece; 2 Pediatric Nephology, Center for Pediatrics and Adolescent Medicine, University of Heidelberg, 69210 Heidelberg, Germany; clauspeter.schmitt@med.uni-heidelberg.de

The peritoneal membrane is the largest internal membrane of the human body, having a surface area that approximates the surface area of the skin. It covers the walls of the abdominal cavity (parietal peritoneum) and the surface of the internal organs (visceral peritoneum) and comprises a monolayer of mesothelial cells, underneath of which there is connective tissue with blood vessels, nerves, lymphatics, and fibroblasts [1,2,3].

The peritoneal membrane has not been sufficiently studied, despite the fact that several pathological conditions are linked to it, namely, the development of abdominal adhesions after surgery, of peritoneal primary mesothelioma and metastasis and of peritonitis and secondary ascites [4,5]. Furthermore, the peritoneum serves as a semipermeable membrane in peritoneal dialysis (PD)—a well-established widespread renal replacement therapy in patients with end-stage renal disease [2]. 

The goal of the Special Issue on “Molecular mechanisms of peritoneal membrane pathophysiology” was to attract original research articles and reviews to broaden our current knowledge on the molecular mediators and mechanisms that govern the response of the peritoneum to pathological infectious and non-infectious stimuli. Molecular signatures, biomarkers, and therapeutic prospects were of particular interest so that a comprehensive overview of peritoneal pathophysiology from different perspectives could be achieved. Experts in peritoneal membrane biology have contributed 10 manuscripts, including 7 original research papers and 3 reviews, that have attracted more than 12,280 views on the *Biomolecules* website (25^th^ of May 2022). 

Nine of the ten manuscripts are related to PD. PD exposes the peritoneum to pathological stimuli, comprising numerous toxins accumulating with chronic kidney disease (CKD), high concentrations of glucose and its derivatives, and treatment-associated infections. The chronic insult induces progressive peritoneal fibrosis, excessive angiogenesis, and mesothelial cell loss. This reduces the dialytic capacity to remove fluid and dissolved toxins and limits long-term use of the life-saving therapy [2,3]. The manuscripts of this Special Issue are focused on the underlying molecular pathomechanisms, on molecular signatures and biomarkers for PD patient status, and on experimental interventions to improve outcomes. This set of highly interesting publications is complemented by an excellent review on the current understanding of post-surgical-related fibrosis. 

Baturina and coworkers describe the mesothelial cell response to osmotic stress as compared to kidney outer medullary collecting duct cells [6]. Each inflow of PD fluid exposes mesothelial cells to far supraphysiological concentrations of glucose required for fluid removal from the patient, and the cellular response to the high osmotic stress is largely unknown. The authors demonstrate both the similarities in regulatory volume decrease mechanisms in peritoneal mesothelial cells when challenged with myo-inositol and urea under hyperosmolar isotonic switch from mannitol as compared with kidney outer medullary-collecting duct cells, and also distinct differences, i.e., a regulatory volume decrease in mesothelial cells but not in kidney outer medullary-collecting duct cells in response to urea. Such an understanding of cellular responses is needed in the search for innovative osmotic agents for PD fluids exerting less local but also less systemic harm. 

Eleftheriadis et al. explored the activation of the General Control Nonrepressible-2 (GCN-2) kinase on primary human peritoneal cells as a means of compromising mesothelial glucotoxicity induced by high glucose concentrations present in PD fluids [7].The GCN-2 activators, halofuginone and tryptophanol, reduced the mesothelial expression of glucose transporters and co-transporters and reduced the cellular glucose uptake and the accumulation of downstream metabolites that lead to advanced glucation end products and increased oxidative stress. The activation of GCN-2 moreover reduced the high glucose-induced mesothelial TGF-β and IL-8 synthesis and of α-SMA, which is a hallmark of mesothelial-to-mesenchymal transition. Since the mesothelium plays a critical role in maintaining peritoneal integrity and function, these results hold promise regarding preservation of the mesothelial monolayer and of the peritoneal membrane function in patients with PD and warrant further investigations in vivo. 

Major PD-associated alterations in plasma fibrinogen glycosylation could be demonstrated by Baralić and colleagues and provide novel insights in the mechanisms of end-stage renal-disease-associated cardiovascular complications. The γ chain of the protein is the most susceptible to disturbing glycosylation, i.e., the chain responsible for the physiological functions of fibrinogen such as coagulation and platelet aggregation. Of particular interest, increasing fibrinogen fucosylation independently predicted ultrafiltration decline and highlights its potential role as a prognostic biomarker in PD.

Three publications addressed therapeutic interventions aiming at a reduction in PD-associated peritoneal damage. The modification of glucose-induced peritoneal membrane transformation was studied by Balzer et al., who investigated in a murine model of PD the effects of SGLT2 inhibition with intraperitoneal administration of dapagliflozin [8]. The authors demonstrated the expression of both SGLT1 and SGLT2 in the peritoneal membrane of mice and humans with normal renal function and on PD and established an inhibitory effect of dapagliflozin on the PD-induced SGLT2 expression increase in the animal model and a reduction in peritoneal fibrosis, angiogenesis and ultrafiltration failure, i.e., of the main drivers of PD modality dropout. Concomitantly, the SGLT-2 inhibition had an immunomodulatory effect. Thus, the authors add another important beneficial aspect of SGLT-2 inhibitors that are widely used in patients with diabetes and studied in CKD and cardiorenal diseases [9].

The protective role of the dipeptide of Alanyl-Glutamine (AlaGln) supplementation in the context of PD was highlighted in two original manuscripts focused on the improvement of the endothelial cell and barrier function [10,11]. In the study of Bartosova et al., the supplementation of AlaGln was tested in human endothelial cell (HUVEC) monolayers and incubated with PD fluids with high and very low concentrations of glucose-degradation products (GDP). Exposed to high GDP PD fluids, AlaGln restored the integrity and paracellular permeability characteristics of the endothelial monolayer by maintaining the abundance of ZO-1 and Claudin-5 along with the clustering of ZO-1 in the cell-to-cell junction at the nanoscale. Supplementation of AlaGln in mice exposed to the same PD fluid also restored arteriolar endothelial claudin-5 abundance, providing further evidence that AlaGln can improve peritoneal sealing and thus presumably the sustainability of PD treatment [10]. 

Compelling evidence on the validity of HUVECs as an in vitro model of PD-induced endothelial effects was provided by Herzog et al. [11]. By means of cross-omics (transcriptomics and proteomics analyses), the authors demonstrated that the gene and protein changes seen in HUVECs incubated with conventional PD fluids overlap to a great extent, with changes seen in arteriolar samples from peritoneal biopsies of PD patients treated with PD fluids rich in GDP. Obvious limitations of the in vitro model as compared to the human ex vivo findings are related to the complex arteriolar tissue structures. Proteomic analyses furthermore revealed the cytoprotective effects of AlaGln, reducing endothelial cellular damage, restoring perturbed abundances of pathophysiologically important proteins and enriching protective biological processes. Overall, the experimental evidence provided in these two studies on AlaGln supports the concept of membrane-preserving additives in PD fluids and justifies further clinical trials. In a randomized controlled clinical trial, AlaGln improved peritoneal membrane semi-permeability, i.e., increased the peritoneal transport of small solutes and reduced peritoneal protein losses [12].

Effluents from the patients participating in the latter trial [12] were used for the study of Grunert et al. [13]. In a different direction, this study tested whether Fourier transform infrared (FTIR) spectroscopy could be used as a means for detecting molecular fingerprints in the effluent of PD patients for the timely detection of PD-related complications. Principal component analysis of the FTIR spectra allowed for the discrimination of effluents at the start and at the end of the PD dwell, as well as for the discrimination of control PD fluids as compared to AlaGln-supplemented PD fluids. The authors combined the molecular fingerprinting of the effluents with clinical data and adopted machine learning approaches that pave the way for further investigations as to whether this proof-of-principle study can diffuse to clinical practice. 

Overall, the seven original studies convincingly demonstrate novel and in-depth understanding of the molecular signaling induced by PD and how this can open the path for therapeutic interventions specifically counteracting detrimental effects exerted by the highly unphysiological PD fluids. They demonstrate the urgent need of an improved PD fluid biocompatibility profile, which thus far to some degree has been achieved in clinical practice with the replacement of glucose by icodextrin for a single long dwell, the reduction in GDP content, and possibly by the introduction of bicarbonate instead of lactate buffer at a neutral to physiological pH. The impact of these improvements on relevant clinical outcome measures, however, is uncertain [14]. In the same direction, adding protective compounds such as AlaGln may be an alternative to mitigate not only local peritoneal but also systemic sequelae of the life-saving PD therapy, but convincing patient benefits still need to be demonstrated.

The reviews of Roumeliotis et al. and of Marchant et al. are focused on two distinct insults imposed by PD treatment to the peritoneal membrane and discussed potential adjuvant or therapeutic approaches [15,16]. The first explored the essential pathophysiological role of increased oxidative stress in the morphological and functional deterioration of the peritoneal membrane and the respective clinical implications. Next to the paramount role of the exceedingly high glucose concentrations and respective derivatives of PD fluids, the authors also summarize the role of further PD fluid’s characteristics, such as acidity and high lactate content, on oxidative-stress-induced peritoneal pathophysiology, but also the role of intercurrent and smoldering infections in PD, especially in the context of high GDP fluids [15]. 

IL-17A has recently been proposed as a potential therapeutic target for chronic inflammatory diseases, including CKD. Marchant et al. focus on the role of IL-17A within the context of PD. Numerous infiltration cells secrete IL-17A and foster peritoneal angiogenesis and mesothelial mesenchymal transition, and sustain inflammatory cell recruitment and inflammation and subsequent fibrosis. IL-17A is centrally involved in PD-induced peritoneal membrane damage and represents a highly promising therapeutic tool [16]. Potential interventions include therapies established in clinical routine such as blockers of the renin angiotensin system, COX-2 inhibitors, and statins, but the evidence is still circumstantial. Experimental studies suggest a role for vitamin D and analogues. The key question to address, however, is whether the blockade of IL-17A by neutralizing antibodies as studied in clinical trials for chronic inflammatory diseases is beneficial in the setting of chronic PD, i.e., whether it preserves peritoneal membrane integrity and—at best—reduces systemic inflammatory and cardiovascular sequelae combined with high tolerability and at reasonable costs. 

The review by Herrick and Wilm comprehensively describes the essential role of the mesothelium in peritoneal homeostasis and in the formation of post-surgical adhesions, a major health and economic burden [17]. They shed new mechanistic light on the mechanisms governing physiological serosal repair and regeneration, considering abdominal site-related specificity, and comprehensively describe the events leading to pathological response following injury, i.e., the plethora of peritoneal cellular and molecular actions, which reflect the complexity of peritoneal homeostatic functions. The authors delineate the limitations of the current experimental approaches and how to overcome these, e.g., by consideration of mesothelial biomolecular marker expression during peritoneal development, and thus provide key information needed to pave the way for urgently needed treatments to limit or even prevent peritoneal adhesion formation. 

Overall, this Special Issue provides great insights into the physiological regulation of the peritoneum and the balanced and pathological response to injurious stimuli, i.e., surgical interventions and PD. Both settings are characterized by limited therapeutic progress and few novel treatments forthcoming to the clinical routine, even though the health-related and economic burden is major and increasing with the growing ageing population. Different specialties with a common interest in peritoneal biology should be brought together to overcome the current barriers of success and apply novel and unpreceded approaches. This Special Issue, albeit focused on PD, illustrates different approaches to common peritoneal diseases and thus represents one step into this direction, hopefully fostering further concerted and urgently needed research activities in the field. 
